# TNF/Ang‐II synergy is obligate for fibroinflammatory pathology, but not for changes in cardiorenal function

**DOI:** 10.14814/phy2.12765

**Published:** 2016-04-28

**Authors:** Magdalena Mayr, Clemens Duerrschmid, Guillermo Medrano, George E. Taffet, Yanlin Wang, Mark L. Entman, Sandra B. Haudek

**Affiliations:** ^1^ Division of Cardiovascular SciencesDepartment of MedicineBaylor College of MedicineHoustonTexas; ^2^ Division of NephrologyDepartment of MedicineBaylor College of MedicineHoustonTexas

**Keywords:** Angiotensin‐II, fibrosis, heart, inflammation, kidney, tumor necrosis factor

## Abstract

Angiotensin‐II (Ang‐II) infusion is associated with the development of interstitial fibrosis in both heart and kidney as a result of chemokine‐dependent uptake of monocytes and subsequent development of myeloid fibroblasts. This study emphasizes on the synergistic role of tumor necrosis factor (TNF) on the time course of Ang‐II‐induced fibrosis and inflammation in heart and kidney. In wild‐type (WT) hearts, Ang‐II‐induced fibrosis peaked within 1 week of infusion and remained stable over a 6‐week period, while the myeloid fibroblasts disappeared; TNF receptor‐1‐knockout (TNFR1‐KO) hearts did not develop a myeloid response or cardiac fibrosis during this time. WT hearts developed more accelerated cardiac hypertrophy and remodeling than TNFR1‐KO. In the kidney, 1‐week Ang‐II infusion did not evoke a fibrotic response; however, after 6 weeks, WT kidneys displayed modest but significant tubulointerstitial collagen deposition associated with the appearance of myeloid cells and profibrotic gene activation. Renal fibrosis was not seen in Ang‐II‐infused TNFR1‐KO. By contrast, while hypertension increased and cardiac function decreased more slowly in TNFR1‐KO than WT, they were equivalently abnormal at 6 weeks. Similarly, serum markers for renal dysfunction were not different after 6 weeks. In conclusion, Ang‐II infusion initiated fibroinflammatory responses with different time courses in heart and kidney, both requiring TNFR1 signaling, and both associated with monocyte‐derived myeloid fibroblasts. TNFR1 deletion obviated the fibroinflammatory effects of Ang‐II, but did not alter changes in blood pressure and cardiorenal function after 6 weeks. Thus, the synergy of TNF with Ang‐II targets the fibroinflammatory component of Ang‐II signaling.

## Introduction

Angiotensin‐II (Ang‐II) is a widely recognized regulator in a broad spectrum of cardiovascular disorders. It plays key roles on several levels in the pathogenesis of heart failure, such as in body fluid homeostasis and hypertension; it also promotes the inflammatory response, induces cardiac hypertrophy, and acts as a profibrotic factor (Sciarretta et al. 2009). Additional studies have suggested a synergistic role for tumor necrosis factor alpha (TNF) in Ang‐II pathophysiology (Sekiguchi et al. 2004; Sriramula et al. 2008; Pellieux et al. 2009). TNF is a major regulator of inflammation and immunity; it is also implicated in affecting cardiac structure and function (Meldrum 1998; Kleinbongard et al. 2010). The actions of TNF are signaled through two distinct TNF receptors, TNFR1 and TNFR2 (Aggarwal 2003). The receptors differ significantly within their intracellular regions, resulting in deleterious as well as protective effects (Cabal‐Hierro and Lazo 2012).

In our previous work, we demonstrated that 1‐week Ang‐II exposure to mice resulted in the sequential and timely regulated uptake of bone marrow‐derived cells into the heart and concurrent induction of proinflammatory and profibrotic factors that drove the maturation of myeloid precursor cells into fibroblasts (Duerrschmid et al. 2015). This development was dependent on monocyte–macrophage chemoattractive protein‐1 (MCP‐1) expression and on TNFR1 signaling (Haudek et al. 2010; Duerrschmid et al. 2013, 2015). To define the TNF/Ang‐II synergy in cardiac fibrosis, we subjected mice deficient in either TNFR1 or TNFR2 to systemic Ang‐II infusion (Duerrschmid et al. 2013). Our results suggested that TNF produced by M1 cells was necessary for mediating a TNFR1‐dependent maturation of M2 cells into fibroblasts. TNFR1‐KO mice were resistant to Ang‐II‐induced cardiac fibrosis and presented less cardiac hypertrophy and left ventricular (LV) remodeling than WT mice, while TNFR2‐KO mice were unaffected (Duerrschmid et al. 2013, 2015). In the first week, TNFR1‐KO mice also displayed better cardiac function and lack of hypertension (Duerrschmid et al. 2013). In the current report, we investigated longer Ang‐II infusion periods.

Parallel to our studies in the heart, our group also characterized the effects of Ang‐II on renal fibrosis (Xu et al. 2011; Xia et al. 2013, 2014a). Using a model of Ang‐II infusion in combination with uninephrectomy, we described a robust chemokine‐dependent uptake of monocytic fibroblast precursors and concurrent development of renal fibrosis, similar as observed in the heart. However, these experiments required a secondary method (uninephrectomy). In the current report, we utilized a model of increased systemic Ang‐II levels in the absence of other interventions and extended the length of infusion to explore the inflammatory and fibrotic events leading to kidney fibrosis and the role of TNF in these mechanisms.

Therefore, the current study sought to define further the cellular and molecular events involved in our proposed TNF/Ang‐II synergy. Specifically, we were interested if TNFR1‐KO mice remained protected from the Ang‐II‐induced hemodynamic changes over time, and how this would influence the development of cardiac and renal fibrosis. Our results indicate that (1) Ang‐II‐induced renal fibrosis not only developed more slowly than cardiac fibrosis, but was also mediated by TNFR1‐dependent mechanisms involving uptake of myeloid fibroblast precursors, and (2) TNFR1‐KO mice remained protected from the development of cardiac and renal fibrosis despite a delayed increase in systolic blood pressure and decrease in cardiorenal function.

## Methods

### Animals

C57BL/6‐Tnfrsf1a^tm1Imx^/J (TNFR1‐KO) and C57BL/6J (WT) mice (8–10 weeks; The Jackson Laboratory, Bar Harbor, ME) were infused with 1.5 *μ*g/kg/min Ang‐II via subcutaneously implanted (inhalational anesthesia with 2% isoflurane via an endotracheal tube) osmotic pumps for up to 6 weeks; control animals received saline (Haudek et al. 2010; Duerrschmid et al. 2013, 2015). All mice were euthanized with 2% isoflurane followed by cervical dislocation. The investigations conformed to the Guide for the Care and Use of Laboratory Animals published by the NIH. All animals were treated in accordance with the guidelines of the Baylor College of Medicine Animal Care and Research Advisory Committee (all procedures were approved by Baylor's Institutional Review Board).

### Tissue staining

Hearts were arrested in diastole, perfusion‐fixed in Zinc‐Tris buffer, and paraffin embedded (Duerrschmid et al. 2015). Areas of collagen deposition as percentages of total myocardial area were evaluated by picrosirius red staining (Duerrschmid et al. 2015). For *α*‐smooth muscle actin (*α*‐SMA) and Mac‐2 evaluation, deparaffinized, dehydrated sections were stained as described previously (Haudek et al. 2010; Duerrschmid et al. 2013). Kidneys were either perfusion fixed and embedded as described for hearts or frozen in Tissue‐Tek^®^ optimal cutting temperature at −80°C, sections were cut from the middle plane. Perfusion‐fixed, rehydrated sections were stained and evaluated for collagen as described for hearts. Frozen sections were assessed for fibrosis by trichrome staining (kit from Diagnostic BioSystems, Pleasanton, CA). Collagen deposition (4 images/section, 2–4 sections/kidney) was evaluated by 2–3 independent, blinded investigators using a scoring system: 0 =  none, 1 = minor, 2 =  modest, 3 =  medium, 4 =  major. For *α*‐SMA and CD68 evaluation, frozen sections were stained as described for heart sections. All tissue sections were mounted in Cytoseal XYL. Bright‐field images were captured on an Olympus CKX41 inverted microscope using QCapturePro. 5.0; ImageJ and ImageProPlus 5.1 were used for quantitative analysis.

### Flow cytometry

Whole hearts and kidneys were excised and cells isolated by enzymatic digestion using 0.1 mg/mL Liberase^TH^ Research Grade (Duerrschmid et al. 2015). Cells were incubated with Calcein^AM^ (Invitrogen Molecular Probes, Carlsbad, CA) to identify live cells (only live cells metabolize calcein to a green‐fluorescent product) together with directly‐conjugated or biotin‐conjugated external antibodies followed by PE‐ or PE‐Cy5‐conjugated secondary antibodies. In separate setups, cells were externally stained, permeabilized, and then incubated with internal antibodies. FITC/PE/PE‐Cy‐5 fluorescence intensities were measured on an EpicsXL‐MCL with companion software. Gating strategies and representative flow diagrams were described earlier in detail (Duerrschmid et al. 2015).

### Quantitative PCR

RNA was isolated from whole frozen tissue, cDNA synthesized, and qPCR performed with iQ SYBR Green Super mix on an CFX96 Touch^™^ thermal cycler (BioRad, Hercules, CA) as described previously (Duerrschmid et al. 2015). Using sequence‐specific primers, relative gene expression was measured by the ΔΔCq method and was normalized to 18S ribosomal RNA levels (Bustin et al. 2009). Primer sequences are summarized in Table 1, some were published previously (Duerrschmid et al. 2015).

**Table 1:** qPCR primers

Gene	Forward primer	Reverse primer
*18s RNA*	cggacaggattgacagattg	caaatcgctccaccaactaa
*α‐SMA*	gctggactctggagatgg	gcagtagtcacgaaggaatag
*CCR2*	gggtcatgatccctatgtgg	tccatgagcagtggtttgaa
*Collagen type I*	gtatgcttgatctgtatctg	cgactcctacatcttctg
*Collagen type III*	gatgaggagccactagactg	gccatcaggaagcacagg
*CXCL16*	acccttgtctcttgcgttcttcct	atgtgatccaaagtaccctgcggt
*Endothelin‐1*	gcaccggagctgagaatgg	gtggcagaagtagacacactc
*IFN‐γ*	actggcaaaaggatggtgac	gacctgtgggttgttgacct
*IL‐1β*	ttgacggaccccaaaagatgaaggg	tccacagccacaatgagtgatactg
*IL‐4*	cctcacagcaacgaagaaca	atcgaaaagcccgaaagagt
*IL‐6*	agttgccttcttgggactga	acaggtctgttgggagtggt
*IL‐13*	gtgtctctccctctgaccct	ggggagtctggtcttgtgtg
*MCP‐1*	tccacaaccacctcaagcacttc	ggcatcacagtccgagtcacac
*Osteopontin*	tgatgacgatgatgatgac	ctcagtccataagccaag
*Periostin*	tggtatcaaggtgctatctgcg	aatgcccagcgtgccataa
*TGF‐β1*	cactggagttgtacggcagtg	agagcagtgagcgctgaatc
*TNF*	ccagtgtgggaagctgtctt	aagcaaaagaggaggcaaca
*TNFR1*	gctgaccctctgctctacgaa	gccatccaccacagcataca
*TNFR2*	tgcgccttgaaaacccattc	ggcacttagagttggggact

5′–3′ forward and reverse primer sequences used for quantitative PCR based on corresponding mouse sequences. Primers were designed and validated according to the MIQE guidelines (Bustin et al. 2009). α‐SMA, a‐smooth muscle actin; CCR2, CC receptor 2; CXCL16, CXC ligand 16; IFN, interferon; IL, interleukin; MCP‐1, monocyte‐macrophage chemoattractive protein‐1; TGF‐b1, transforming growth factor b1; TNF, tumor necrosis factor alpha; TNFR1 or 2, TNF receptor 1 or 2.

### ELISA

Blood was collected upon euthanization and allowed to coagulate at room temperature before centrifugation; serum was aliquoted and frozen at −80°C. Blood urea nitrogen (BUN) and creatinine levels were measured using commercially available kits (Sigma‐Aldrich, St. Louis, MO) on a VersaMax Spectrophotometer (Molecular Devices, Sunnyvale, CA).

### Cardiovascular measurements

Anatomic parameters were obtained by 2D‐directed M‐mode echocardiography (Vevo770; Visual Sonics, Bothell, WA), functional parameters by Doppler Ultrasound (Model DSPW, Indus Instruments, Webster, TX) as described previously (Duerrschmid et al. 2013). Data were stored and analyzed offline. Blood pressure measurements were obtained by the tail‐cuff method (VisitechBP2000, Visitech Systems, Apex, NC) (Duerrschmid et al. 2013).

### Statistical analysis

**Figure 1:**
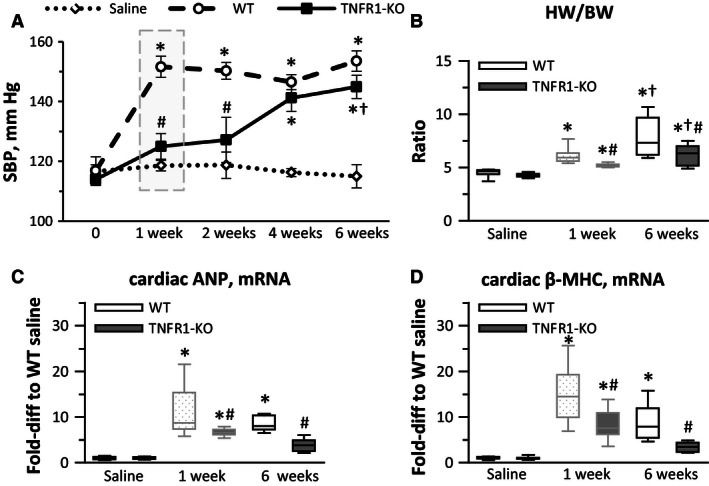
TNFR1‐KO mice were not protected from 6‐week Ang‐II‐induced hypertension, but development of cardiac hypertrophy was reduced. (A) Baseline values of systolic blood pressure (SBP) were obtained before treatment (“0”) and repeated at the indicated times during Ang‐II treatment (saline group: *n* = 6 [3× wild‐type (WT) and 3× TNFR1‐KO were pooled], WT group: *n* = 6, TNFR1‐KO group: *n* = 8). (B) Heart weight‐to‐body weight (HW/BW) ratio (*n* = 10–18). (C and D) Transcriptional activation of cardiac atrial natriuretic peptide (ANP) and *β*‐myosin heavy chain (*β*‐MHC) (*n* = 8/group). *P < 0.05 between saline‐ and Ang‐II‐treated groups of the same genotype. ^†^ *P* < 0.05 between 1‐ and 6‐week Ang‐II‐treated groups of the same genotype. ^#^ *P* < 0.05 between WT and TNFR1‐KO groups at the same time point. Data for 1‐week time points were previously published (Duerrschmid et al. 2013), but are shown in faded gray for direct comparisons.

All results are expressed as mean ± SEM. Kolmogorov–Smirnov tests were used to evaluate normal distribution. To compare differences between >2 groups, one‐way ANOVA followed by Tukey–Kramer post hoc testing (parametric) or Kruskal–Wallis followed by Dunn's post hoc testing (nonparametric) were used. To compare differences between two groups, unpaired two‐tailed *t* tests (parametric) or Mann–Whitney tests (nonparametric) were used; additionally in Tables 2 and 3, paired *t*‐tests (parametric) or Wilcoxon rank‐sum tests (nonparametric) were used. In Figure 1A, repeated measures ANOVA followed by Tukey–Kramer post hoc testing was used. A *P *<* *0.05 was considered statistically significant (GraphPad InStat 3.06).

**Table 2:** Cardiac anatomical parameters

	WT	WT	WT	TNFR1‐KO	TNFR1‐KO	TNFR1‐KO
	baseline	6 weeks	% change	baseline	6 weeks	% change
Global parameters						
*n*	12	12	12	13	13	13
* *BW, g	25.3 ± 1.6	25.6 ± 1.3	1.5 ± 4.1	21.5 ± 1.8^b^	22.4 ± 1.7^a^^,^ b	4.6 ± 6.2
Cardiac echocardiographic parameters						
LVEDD, mm	3.98 ± 0.07	4.35 ± 0.15^a^	9.3 ± 3.5	3.78 ± 0.08	3.89 ± 0.11^b^	2.8 ± 1.7^c^
LVEDV, mm^3^	69.6 ± 3.0	87.0 ± 6.7^a^	25.6 ± 8.7	61.7 ± 3.0	66.3 ± 4.3^b^	7.2 ± 4.0^c^
LVESD, mm	3.05 ± 0.07	3.27 ± 0.14	7.1 ± 4.3	2.85 ± 0.09	2.72 ± 0.15^b^	5.2 ± 2.9^c^
LVESV, mm^3^	36.9 ± 2.1	40.7 ± 5.0	12.2 ± 14.6	30.4 ± 3.0	29.3 ± 3.9	0.5 ± 12.6
Fractional shortening, %	23.3 ± 1.3	29.8 ± 3.1	31.0 ± 14.3	26.7 ± 2.1	30.4 ± 2.5	15.3 ± 6.3
Ejection fraction, %	46.9 ± 2.3	55.6 ± 4.1	20.8 ± 9.5	52.2 ± 3.2	57.4 ± 3.6	10.9 ± 4.6
Stroke volume, *μ*L	32.7 ± 2.2	46.4 ± 3.1^a^	45.1 ± 9.7	31.4 ± 1.3	37.0 ± 2.2^a^, ^b^	18.0 ± 5.4^c^
LVPW‐d, mm	0.74 ± 0.02	0.88 ± 0.06^a^	20.0 ± 6.7	0.71 ± 0.04	0.72 ± 0.04^b^	2.6 ± 4.5^c^
LVPW‐s, mm	1.02 ± 0.04	1.21 ± 0.13	16.6 ± 8.6	0.99 ± 0.05	1.00 ± 0.05	1.2 ± 4.1
LVAW‐d, mm	0.66 ± 0.02	0.84 ± 0.02^a^	27.8 ± 5.1	0.70 ± 0.01	0.77 ± 0.03^a^	10.7 ± 3.7^c^
LVAW‐s, mm	0.90 ± 0.03	1.23 ± 0.05^a^	38.7 ± 6.9	0.96 ± 0.03	1.13 ± 0.05^a^	17.9 ± 4.1^c^
LV mass, mg	78.1 ± 2.7	119.4 ± 7.0^a^	52.2 ± 5.3	72.8 ± 4.3	83.0 ± 4.0^a^^,^ b	16.8 ± 6.6^c^

Six‐week Ang‐II infusion induced LV remodeling in wild‐type (WT) mice, but these changes were significantly smaller in TNFR1‐KO mice. The % change was calculated within the same mouse with parameters measured before (baseline) and after 6‐week Ang‐II infusion.

BW, body weight, LVEDD and LVESD, left ventricular end diastolic/systolic diameter, LVEDV and LVESV, left ventricular end diastolic/systolic volume, LVPW and LVAW, left ventricular posterior/anterior wall thickness, ‐d/‐s: during diastole/systole.

a *P* < 0.05 between baseline and 6‐week Ang‐II‐treated groups of the same genotype (paired tests).

b *P* < 0.05 between WT and TNFR1‐KO groups at the same time point (unpaired tests).

c *P* < 0.05 between the “% change” groups.

**Table 3:** Cardiac functional parameters

	WT	WT	WT	TNFR1‐KO	TNFR1‐KO	TNFR1‐KO
	baseline	6 weeks	% change	baseline	6 weeks	% change
Global parameters						
*n*	12	12	12	13	13	13
HR, bpm	437 ± 51	532 ± 76^a^	25.0 ± 30.4	421 ± 38	510 ± 85^a^	21.3 ± 16.5
Cardiac Doppler parameters						
E‐lin dec time, msec	46.3 ± 16.6	29.7 ± 12.9^a^	31.2 ± 29.1	38.1 ± 18.3	35.1 ± 19.2	16.5 ± 93.9
E‐peak vel, cm/sec	79.9 ± 9.2	78.6 ± 16.9	−0.4 ± 24.7	75.4 ± 11.2	75.9 ± 6.7	0.3 ± 14.9
A‐peak vel, cm/sec	107 ± 16	146 ± 12^a^	39.1 ± 22.9	92 ± 14^b^	127 ± 36^a^	37.3 ± 31.6
Pre‐EjT, msec	15.8 ± 2.3	16.7 ± 2.4	8.4 ± 24.4	17.6 ± 2.4	18.3 ± 2.0	5.8 ± 18.5
Pre‐EjT/EjT	0.31 ± 0.04	0.38 ± 0.07^a^	24.1 ± 23.8	0.34 ± 0.05	0.41 ± 0.05^a^	21.3 ± 22.1
Tei index	0.58 ± 0.07	0.81 ± 0.15^a^	42.1 ± 31.6	0.61 ± 0.09	0.81 ± 0.15^a^	34.1 ± 26.1

Six‐week Ang‐II infusion induced cardiac dysfunction in wild‐type (WT) and in TNFR1‐KO mice to a similar extent. The % change was calculated within the same mouse with parameters measured before (baseline) and after 6‐week Ang‐II infusion.

HR, heart rate; E‐lin dec time, E‐linear deceleration time; vel, velocity; EjT, ejection time; Tei index, myocardial performance index (a heart rate independent time interval index that combines both systolic and diastolic cardiac performance) was calculated as [isovolumetric relaxation time + isovolumetric contraction time]/EjT.

a *P* < 0.05 between baseline and 6‐week Ang‐II‐treated groups of the same genotype (paired tests).

b *P* < 0.05 between WT and TNFR1‐KO groups at the same time point (unpaired tests). There was no statistical difference between the “% change” groups.

## Results

### Six‐week infusion of Ang‐II into WT and TNFR1‐KO mice resulted in the same degree of hypertension and cardiac dysfunction; by contrast, cardiac hypertrophy and remodeling remained lower in TNFR1‐KO

We previously reported that TNFR1‐KO mice were protected from the development of hypertension, cardiac hypertrophy, remodeling, and dysfunction after 1‐week Ang‐II infusion (Duerrschmid et al. 2013). We now found that systolic blood pressure in TNFR1‐KO mice increased between 2 and 4 weeks of Ang‐II infusion to levels not different than WT levels (Fig. 1A), indicating that the lack of TNFR1 signaling delayed, but did not prevent the development of hypertension. By contrast, the development of cardiac hypertrophy remained significantly lower in TNFR1‐KO hearts compared to WT hearts. As shown in Figure 1B–D, the ratio of heart weight to body weight was higher in Ang‐II‐infused WT than in TNFR1‐KO hearts, as was the transcriptional expression of atrial natriuretic peptide (ANP) and *β*‐myosin heavy chain (*β*‐MHC), two hypertrophy‐related genes, although all three parameters increased over baseline in TNFR1‐KO hearts. We extended these observations by evaluating cardiovascular parameters using echocardiography and Doppler ultrasound. We found that exposure to Ang‐II worsened LV remodeling in WT mice. However, TNFR1‐KO mice displayed less ventricular remodeling as evidenced by lower wall thickness and preserved LV dimensions, resulting in significantly lower LV mass in Ang‐II‐treated TNFR1‐KO hearts compared to WT hearts (Table 2). Yet, similar to the development of hypertension, changes in diastolic and systolic parameters in TNFR1‐KO mice were not different from WT changes after 6‐week Ang‐II infusion (Table 3).

### In WT hearts, 6‐week infusion of Ang‐II maintained fibrosis, but did not induce fibrosis in TNFR1‐KO hearts

**Figure 2:**
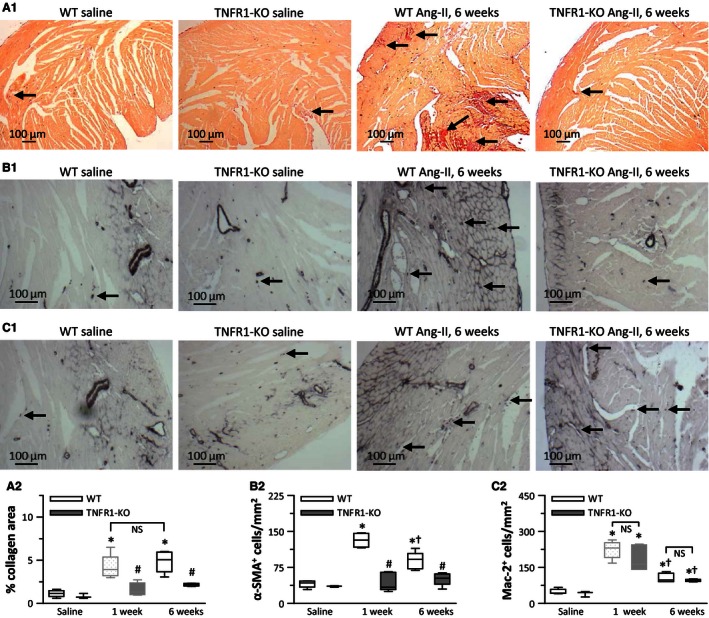
Cardiac fibrosis was maintained during 6‐week Ang‐II infusion in wild‐type (WT), but did not develop in TNFR1‐KO mice. Perfusion‐fixed tissue sections were stained with (A) picrosirius red to determine interstitial collagen deposition, or with antibodies detecting (B) *α*‐smooth muscle actin (*α*‐SMA; myofibroblast marker) and (C) Mac‐2 (macrophage marker) expressing cells. Representative images of saline and 6‐week Ang‐II stains are shown above quantitative data that also include 1‐week treatment groups (*n* = 6/group, except TNFR1‐KO saline: *n* = 3). *P < 0.05 between saline‐ and Ang‐II‐treated groups of the same genotype. ^†^ *P* < 0.05 between 1‐ and 6‐week Ang‐II‐treated groups of the same genotype. ^#^ *P* < 0.05 between WT and TNFR1‐KO groups at the same time point. NS: no significant difference. Data for 1‐week collagen area and Mac‐2 count were previously published (Duerrschmid et al. 2013), but are shown in faded gray for direct comparisons.

We previously characterized the Ang‐II‐induced cardiac fibrosis after 1 week of infusion (Duerrschmid et al. 2013, 2015). To determine if the observed increase in systolic blood pressure resulted in the development of cardiac fibrosis, we exposed TNFR1‐KO mice to Ang‐II for 6 weeks. As shown in Figure 2A1 (qualitatively) and A2 (quantitatively), WT hearts displayed increased interstitial collagen deposition at similar levels as for 1 week, indicating that cardiac fibrosis was maintained over time. By contrast, Ang‐II‐treated TNFR1‐KO hearts still did not show areas of collagen deposition after 6‐week infusion. These observations were supported by the increased presence of *α*‐SMA^+^ myofibroblasts in WT, albeit at lower levels than observed after 1 week, but not in TNFR1‐KO mice (Fig. 2B1 and B2).The overall influx of macrophages in response to Ang‐II did not differ between groups, as the amounts of Mac‐2^+^ cells in 6‐week Ang‐II exposed WT and TNFR1‐KO hearts were similar, although both levels were significantly lower than observed after 1‐week Ang‐II infusion (Fig. 2C1 and C2).

### In WT hearts, 6‐week infusion of Ang‐II sustained increased transcriptional activation of profibrotic factors, but not of proinflammatory factors; TNFR1‐KO hearts remained protected

In earlier reports, we described the temporal profile of transcriptional upregulation of inflammation, fibrosis, and lymphokine‐related genes after 1‐week Ang‐II exposure (Duerrschmid et al. 2013, 2015). We now evaluated their expression at 6 weeks of Ang‐II treatment (Table 4). We found that in WT hearts, fibrosis‐related genes (collagen types I and III, *α*‐SMA, transforming growth factor [TGF]‐*β*1) were significantly upregulated compared to saline‐treated hearts, albeit at lower levels than after 1‐week Ang‐II infusion. By contrast, proinflammatory interleukin (IL)‐1*β*, IL‐6, and interferon (IFN)‐*γ* transcription was several folds lower than after 1‐week Ang‐II infusion, and proinflammatory TNF and MCP‐1 transcription was not upregulated at all, indicating that proinflammatory transcription dissipated over time. In TNFR1‐KO hearts, all profibrotic and proinflammatory factors measured were not significantly different after 6‐week Ang‐II infusion from saline‐treated hearts. Although 6‐week data for profibrotic IL‐13 did not reach statistical significance, IL‐13 levels were slightly elevated in both WT and TNFR1‐KO hearts at the 6‐week time point, confirming our previous observations that lymphokine‐related factors were not affected by TNFR1 deficiency (Duerrschmid et al. 2015).

**Table 4:** Transcriptional gene activation in heart

Gene name	WT	WT	WT	TNFR1‐KO	TNFR1‐KO	TNFR1‐KO
	saline	1 week^a^	6 weeks	saline	1 week^a^	6 weeks
	*n* = 8	*n = 8*	*n* = 8^b^	*n* = 8	*n = 8*	*n* = 8^b^
	Data represent fold‐increase over WT saline					
Fibrosis‐related factors						
*Collagen type I*	1.0 ± 0.1	9.0 ± 1.0^c^	3.6 ± 0.7^c^^,^ d	1.2 ± 0.2	4.1 ± 0.7^c^^,^ e	1.4 ± 0.1^d^
*Collagen type III*	1.0 ± 0.1	9.4 ± 1.0^c^	4.8 ± 0.7^c^^,^ d	1.2 ± 0.1	4.4 ± 0.4^c^^,^ e	1.9 ± 0.2^d^^,^ e
*α‐SMA*	1.0 ± 0.1	4.4 ± 0.5^c^	2.1 ± 0.3^c^^,^ d	1.0 ± 0.1	2.1 ± 0.2^c^^,^ e	1.0 ± 0.1^d^
*TGF‐β1*	1.0 ± 0.1	3.6 ± 0.6^c^	2.2 ± 0.2^c^^,^ d	1.1 ± 0.1	1.5 ± 0.1^e^	1.0 ± 0.1^e^
*Osteopontin*	1.0 ± 0.1	47 ± 13^c^	3.3 ± 0.4^d^	1.1 ± 0.3	8.0 ± 1.7^e^	3.0 ± 0.6
*Periostin*	1.0 ± 0.1	34 ± 4^c^	4.9 ± 0.6^d^	1.3 ± 0.1	18 ± 2^c^, ^e^	1.7 ± 0.2
*Endothelin‐1*	1.0 ± 0.1	3.3 ± 0.4^c^	1.7 ± 0.2^d^	1.6 ± 0.1	1.7 ± 2.0^e^	1.4 ± 0.3
Inflammatory cytokines, chemokines, and their receptors						
*TNF*	1.0 ± 0.1	2.9 ± 0.2^c^	1.4 ± 0.2^d^	1.2 ± 0.1	1.5 ± 0.1^e^	1.7 ± 0.4
*TNFR1*	1.0 ± 0.1	1.5 ± 0.2	1.3 ± 0.2	n/a	n/a	n/a
*TNFR2*	1.0 ± 0.0	2.7 ± 0.3^c^	1.2 ± 0.1^d^	1.2 ± 0.1	2.3 ± 0.4^c^	1.1 ± 0.1^d^
*MCP‐1*	1.0 ± 0.1	4.7 ± 0.6^c^	1.4 ± 0.1^d^	0.9 ± 0.1	1.2 ± 0.1^e^	1.0 ± 0.3
*CCR2*	1.0 ± 0.1	11 ± 2^c^	2.0 ± 0.3^d^	1.0 ± 0.2	5.4 ± 0.7^c^^,^ e	1.9 ± 0.3
*IL‐1β*	1.0 ± 0.1	7.2 ± 1.3^c^	2.8 ± 0.4^d^	1.2 ± 0.2	2.0 ± 0.5^e^	1.2 ± 0.1
*IL‐6*	1.0 ± 0.1	22 ± 4^c^	6.1 ± 1.1^d^	1.2 ± 0.3	7.4 ± 1.4^e^	2.4 ± 0.3
Th1‐ and Th2‐related lymphokines						
*IFN‐γ*	1.0 ± 0.2	4.8 ± 0.6^c^	1.9 ± 0.2^d^	1.3 ± 0.2	4.7 ± 0.4^c^	2.0 ± 0.2^d^
*IL‐4*	1.0 ± 0.0	4.2 ± 0.9^c^	1.7 ± 0.3^d^	1.0 ± 0.2	4.3 ± 0.5^c^	1.5 ± 0.4^d^
*IL‐13*	1.0 ± 0.1	4.6 ± 1.2^c^	2.3 ± 0.6	1.0 ± 0.2	5.1 ± 1.0^c^	1.9 ± 0.8

In wild‐type (WT), hearts, after 6‐week Ang‐II infusion, transcriptional activities of profibrotic genes remained upregulated, while proinflammatory factors dissipated; TNFR1‐KO mice stayed protected (see Table 1 for abbreviations).

a Data for 1 week (except for periostin and endothelin‐1) were previously published (Duerrschmid et al.^2013^,^2015^), but are listed here for direct comparisons.

b *n* = 6 for 6‐week data on IL‐4 and IL‐13.

c *P* < 0.05 between saline‐ and Ang‐II‐treated groups of the same genotype.

d *P* < 0.05 between 1‐ and 6‐week Ang‐II‐treated groups of the same genotype.

e *P* < 0.05 between WT and TNFR1‐KO groups at the same time point.

### In both WT and TNFR1‐KO hearts, M1, M2, and fibroblast precursor cells were absent after 6‐week Ang‐II infusion

**Figure 3:**
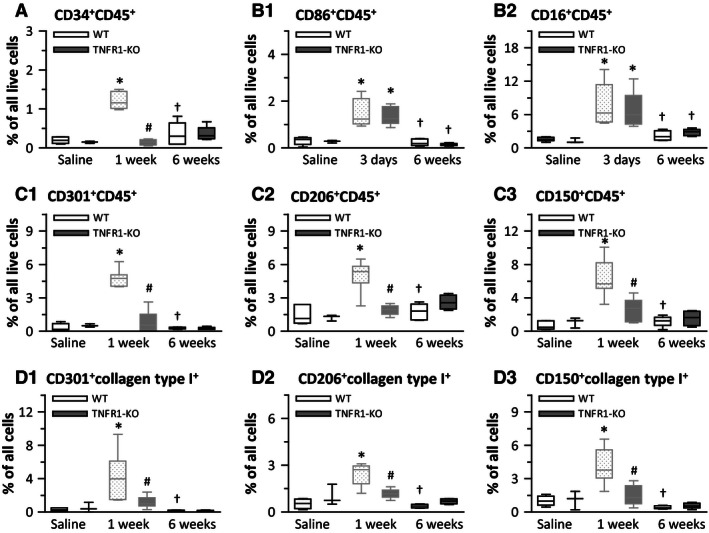
Monocytic precursor cells were absent in both wild‐type (WT) and TNFR1‐KO hearts after 6‐week Ang‐II infusion. Isolated cells from whole hearts were subjected to flow cytometry (see (Duerrschmid et al. 2015) for gating strategies and representative flow diagrams). For external marker detection, live cells were identified via calcein uptake (*n* = 6/group, except for TNFR1‐KO saline *n* = 3). *P < 0.05 between saline‐ and Ang‐II‐treated groups within the same genotype. ^†^ *P* < 0.05 between 1‐week (3 days) and 6‐week Ang‐II‐treated groups of the same genotype. ^#^ *P* < 0.05 between WT and TNFR1‐KO groups at the same time point. Data for 1‐week/3‐day time points were previously published (Duerrschmid et al. 2015), but are shown in faded‐gray for direct comparisons.

We previously characterized the Ang‐II‐induced cellular uptake after 3 and 7 days of infusion (Duerrschmid et al. 2013, 2015). After 6 weeks of Ang‐II treatment, we did not find increased numbers of CD34^+^CD45^+^ fibroblast precursor cells (Fig. 3A), of CD86^+^CD45^+^ and CD16^+^CD45^+^ proinflammatory M1 cells (Fig. 3B1 and B2), of CD301^+^CD45^+^, CD206^+^CD45^+^, and CD150^+^CD45^+^ profibrotic M2 cells (Fig. 3C1, C2, and C3), or of collagen‐expressing M2 cells (Fig. 3D1, D2, and D3) in WT hearts or in TNFR1‐KO hearts. These data are in agreement with our hypothesis that as Ang‐II‐induced cardiac MCP‐1 dissipates (Table 4), myeloid cells in the heart are absent as well. To support these data, we separated CD45^high^ (monocyte‐derived marker) from CD45^low^ (tissue‐resident marker) expressing cells. We found that in WT hearts, the levels of CD45^high^ expressing cells were maximal after 1‐week Ang‐II infusion, but returned to baseline by 6 weeks (saline: 3.3 ± 0.8, 1‐week: 11.0 ± 1.4, 6‐weeks: 3.0 ± 0.2% of all live cells, *n* = 7/group). By contrast, levels of CD45^low^ expressing cells did not change over time (saline: 3.3 ± 0.8, 1 week: 3.9 ± 1.1, 6 weeks: 3.9 ± 1.6% of all live cells, *n* = 7/group).

### In WT kidneys, 6‐week infusion of Ang‐II induced fibrosis, but not in TNFR1‐KO kidneys

**Figure 4:**
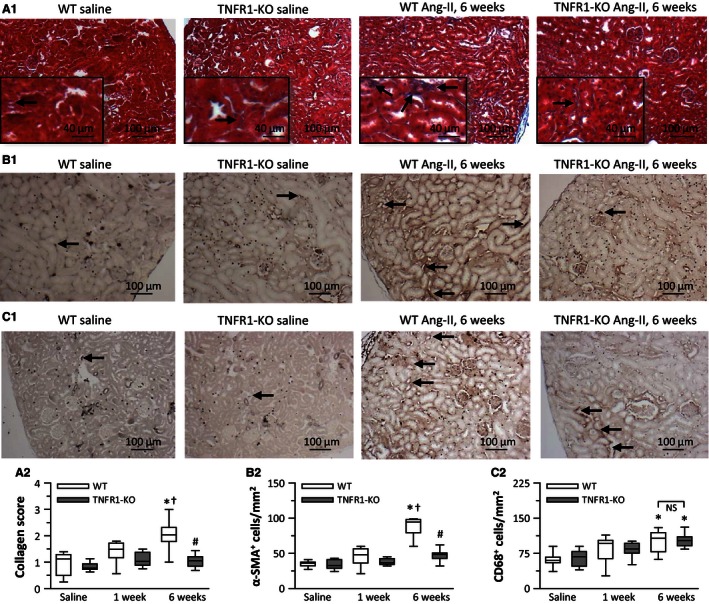
Ang‐II induced the development of renal fibrosis after 6‐week infusion in wild‐type (WT), but not in TNFR1‐KO mice. Frozen tissue sections were stained with (A) trichrome to determine tubulointerstitial collagen deposition, or with antibodies detecting (B) *α*‐smooth muscle actin (*α*‐SMA; myofibroblast marker) and (C) CD68 (macrophage marker) expressing cells. Representative images of saline and 6‐week Ang‐II stains are shown above quantitative data that also include 1‐week treatment groups (*n* = 8/group). *P < 0.05 between saline‐ and Ang‐II‐treated groups of the same genotype. ^†^ *P* < 0.05 between 1‐ and 6‐week Ang‐II‐treated groups of the same genotype. ^#^ *P* < 0.05 between WT and TNFR1‐KO groups at the same time point. NS, no significant difference.

To study the development of renal fibrosis in response to Ang‐II, we exposed WT and TNFR1‐KO mice to Ang‐II without other interventions. As shown in Figure 4, in WT kidneys we did not observe histological changes after 1 week of infusion. However, after 6 weeks, we noted modest, but significant extracellular matrix deposition and cellular infiltrates. Specifically, at 6 weeks we found increased tubulointerstitial collagen deposition in WT mice (Fig. 4A1 and A2). Because the increase in fibrosis was small, we used two different methodological approaches for evaluation. Figure 4A1 shows representative trichrome staining of frozen kidney sections, and A2 the quantitative analysis using a scoring system from 0 to 4 with “0” indicating no collagen and “4” indicating major collagen deposition. We found that collagen deposition roughly doubled in WT kidneys after 6‐week Ang‐II infusion, but not in TNFR1‐KO kidneys. We confirmed these observations by staining perfusion‐fixed tissue sections with picrosirius red and quantitation of the collagen area (WT saline: 0.9 ± 0.1%, WT 1 week: 1.0 ± 0.1%, WT 6 weeks: 1.7 ± 0.1%, and TNFR1‐KO 6 weeks: 0.8 ± 0.1%; *n* = 6–8/group). Of note, the development of Ang‐II‐induced tubulointerstitial collagen deposition in TNFR2‐KO kidneys did not differ from WT levels (trichrome staining score: saline: 0.7 ± 0.1, 1 week: 1.1 ± 0.1, 6 weeks: 2.0 ± 0.4, *n* = 6/group). Six‐week Ang‐II infusion also increased the number of *α*‐SMA^+^ cells in WT kidneys, and those were absent in TNFR1‐KO kidneys (Fig. 4B1 and B2). Similar to our observations in the heart, the overall influx of macrophages in response to Ang‐II did not differ between groups, as the amount of CD68^+^ cells increased over 6‐week Ang‐II exposure in both WT and TNFR1‐KO kidneys (Fig. 4C1 and C2). Taken together, 6‐week exposure of Ang‐II induced renal fibrosis, as evidenced by tubulointerstitial collagen deposition and the presence of myofibroblasts in WT, but not in TNFR1‐KO kidneys.

### In WT kidneys, 6‐week infusion of Ang‐II induced an increase of proinflammatory and profibrotic factors, but not in TNFR1‐KO kidneys

We evaluated upregulation of distinct inflammatory and fibrotic factors in the kidney after Ang‐II infusion. Table 5 summarizes our results. We found that transcriptional activation of fibrosis‐related genes (collagen types I and III, *α*‐SMA, TGF‐*β*1, periostin, endothelin‐1) in WT kidneys were not increased after 1‐week infusion, but significantly increased after 6‐week Ang‐II infusion. Similarly, factors associated with inflammation (TNF, IL‐1*β*, IL‐6) were increased at 6 weeks. Importantly, expression of proinflammatory MCP‐1 and its receptor CCR2 were highly upregulated; by contrast, CXCL16 was not upregulated in our model. Ang‐II infusion in TNFR1‐KO mice did not increase the transcriptional expression of inflammation and fibrosis‐related genes in the kidney, with the exception of IL‐13, which was equally upregulated in WT and TNFR1‐KO kidneys after 6‐week infusion. This confirmed our observations in the heart, which was that expression of Th1‐ and Th2‐related lymphokines was independent of TNFR1 signaling.

**Table 5:** Transcriptional gene activation in kidney

Gene name	WT	WT	WT	TNFR1‐KO	TNFR1‐KO	TNFR1‐KO
	saline	1 week	6 weeks	saline	1 week	6 weeks
	*n* = 8	*n* = 8	*n* = 8	*n* = 8	*n* = 8	*n* = 8
	Data represent fold‐increase over WT saline					
Fibrosis‐related factors						
*Collagen type I*	1.0 ± 0.1	1.7 ± 0.4	2.4 ± 0.1^b^	1.1 ± 0.2	1.2 ± 0.2	1.1 ± 0.2^b^
*Collagen type III*	1.0 ± 0.1	1.5 ± 0.3	2.5 ± 0.2^b^^,^ a	1.0 ± 0.2	1.3 ± 0.2	1.1 ± 0.2^b^
*α‐SMA*	1.0 ± 0.1	1.8 ± 0.2	3.6 ± 0.2^b^^,^ a	1.1 ± 0.1	1.7 ± 0.2	1.9 ± 0.2^b^
*TGF‐β1*	1.0 ± 0.1	1.2 ± 0.1	1.9 ± 0.2^b^^,^ a	1.1 ± 0.1	1.1 ± 0.1	1.1 ± 0.1^b^
*Osteopontin*	1.0 ± 0.1	1.6 ± 0.4	2.0 ± 0.1	1.1 ± 0.1	1.4 ± 0.2	1.9 ± 0.4
*Periostin*	1.0 ± 0.1	1.6 ± 0.3	2.4 ± 0.2^b^	0.9 ± 0.2	1.4 ± 0.2	1.3 ± 0.2^b^
*Endothelin‐1*	1.0 ± 0.1	1.4 ± 0.3	3.4 ± 0.8^b^^,^ a	0.9 ± 0.1	0.7 ± 0.2	1.5 ± 0.2^b^
Inflammatory cytokines, chemokines, and their receptors						
*TNF*	1.0 ± 0.1	1.6 ± 0.2	13 ± 4^b^^,^ a	0.9 ± 0.1	1.0 ± 0.2	3.6 ± 1.1^b^
*TNFR1*	1.0 ± 0.1	1.2 ± 0.1	1.2 ± 0.2	n/a	n/a	n/a
*TNFR2*	1.0 ± 0.1	1.2 ± 0.2	1.3 ± 0.1	1.1 ± 0.2	0.8 ± 0.1	1.5 ± 0.4
*MCP‐1*	1.0 ± 0.2	1.4 ± 0.2	2.3 ± 0.2^b^^,^ a	0.9 ± 0.2	0.8 ± 0.2	1.0 ± 0.2^b^
*CCR2*	1.0 ± 0.2	1.1 ± 0.1	2.6 ± 0.3^b^^,^ a	1.0 ± 0.2	1.0 ± 0.2	1.3 ± 0.2^b^
*CXCL16*	1.0 ± 0.1	1.3 ± 0.1	1.4 ± 0.1	1.4 ± 0.1	1.1 ± 0.1	1.1 ± 0.1
*IL‐1β*	1.0 ± 0.1	1.5 ± 0.2	2.1 ± 0.2^b^	1.2 ± 0.2	1.2 ± 0.3	1.1 ± 0.2^b^
*IL‐6*	1.0 ± 0.1	2.0 ± 0.6	3.8 ± 1.2^b^	1.0 ± 0.1	1.5 ± 0.3	1.1 ± 0.1^b^
Th1‐ and Th2‐related lymphokines						
*IFN‐γ*	1.0 ± 0.2	0.6 ± 0.0	0.8 ± 0.1	1.0 ± 0.2	0.6 ± 0.1	0.6 ± 0.2
*IL‐4*	1.0 ± 0.1	1.1 ± 0.1	1.4 ± 0.3	1.2 ± 0.2	1.3 ± 0.5	0.7 ± 0.2
*IL‐13*	1.0 ± 0.1	1.9 ± 0.3	4.1 ± 1.4^b^	0.9 ± 0.1	1.9 ± 0.4	3.7 ± 1.1^b^

In wild‐type (WT) kidneys, transcriptional activities of proinflammatory and profibrotic factors were upregulated after 6‐week Ang‐II infusion, but not in TNFR1‐KO kidneys. (see table 1 for abbreviations)

*P* < 0.05 between saline‐ and Ang‐II‐treated groups of the same genotype.

a *P* < 0.05 between 1‐ and 6‐week Ang‐II‐treated groups of the same genotype.

b *P* < 0.05 between WT and TNFR1‐KO groups at the same time point.

### In WT kidneys, 6‐week infusion of Ang‐II induced the presence of M1, M2, and fibroblast precursor cells, but not in TNFR1‐KO kidneys

**Figure 5:**
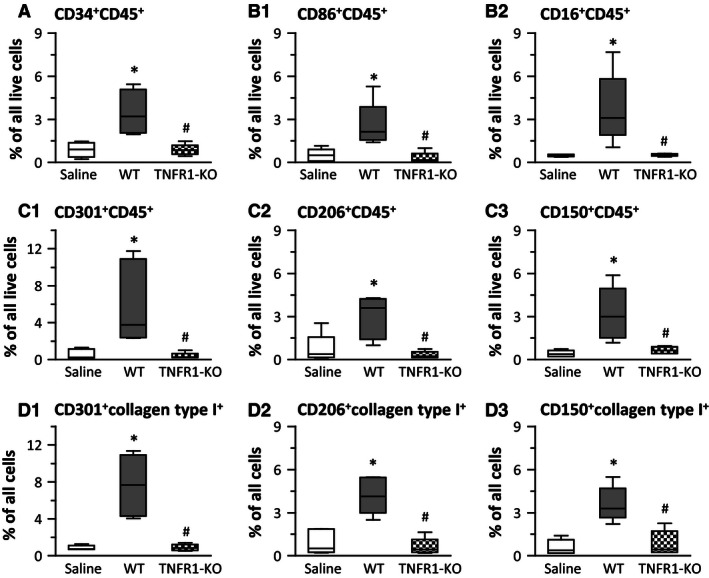
Ang‐II induced the appearance of monocytic precursor cells in wild‐type (WT) kidneys after 6‐week infusion, but not in TNFR1‐KO kidneys. Isolated cells from whole kidneys were subjected to flow cytometry (see (Duerrschmid et al. 2015) for gating strategies and representative flow diagrams). For external marker detection, live cells were identified via calcein uptake (*n* = 6/group; for saline: 3× WT and 3× TNFR1‐KO were pooled). *P < 0.05 between saline‐ and Ang‐II‐treated groups. ^#^ *P* < 0.05 between WT and TNFR1‐KO groups. Pilot studies using *n* = 4 WT mice to test for cellular influx at 1‐week Ang‐II infusion showed no presence of monocytic cells in the kidney (data not shown).

Because MCP‐1 was upregulated in WT kidneys after 6‐week Ang‐II infusion, but not after 1 week (Table 5), we investigated the presence of these cells in the 6‐week Ang‐II exposed kidney. We found increased numbers of CD34^+^CD45^+^ fibroblast precursor cells (Fig. 5A), of CD86^+^CD45^+^ and CD16^+^CD45^+^ proinflammatory M1 cells (Fig. 5B1 and B2), and of CD301^+^CD45^+^, CD206^+^CD45^+^, and CD150^+^CD45^+^ profibrotic M2 cells (Fig. 5 C1, C2, and C3) in WT kidneys. Further analysis revealed that M2 cells were also positive for collagen type I expression (Fig. 5D1, D2, and D3). These cell populations were all absent in the Ang‐II‐exposed TNFR1‐KO kidney, including a lack of proinflammatory M1 cells which was in contrast to our findings in the heart in which M1 cells were highly present in the Ang‐II‐challenged TNFR1‐KO heart.

### Six‐week infusion of Ang‐II decreased renal function to the same degree in both WT and TNFR1‐KO mice

**Figure 6:**
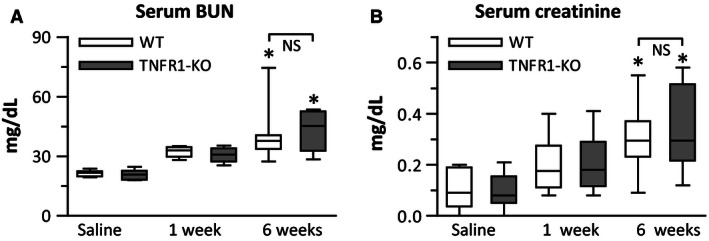
Ang‐II induced renal failure after 6‐week infusion in both WT and TNFR1‐KO mice: Serum concentrations of (A) blood urea nitrogen (BUN;* n* = 10/group) and (B) creatinine (*n* = 8/group). *P < 0.05 between saline‐ and Ang‐II‐treated groups of the same genotype. NS, no significant difference.

To evaluate renal function, we measured serum levels of BUN and creatinine. We found that levels of both markers significantly increased in both WT and TNFR1‐KO mice to a similar extent after 6‐week Ang‐II exposure (Fig. 6A and B), indicating a decline in renal function independent of TNFR1 signaling.

## Discussion

Our laboratory has studied the role of TNF in Ang‐II‐induced cardiac pathology and of Ang‐II in renal dysfunction. The current study is an extension of our previous observations characterizing the time course of the inflammatory and fibrotic effects of Ang‐II on heart and kidney and its relationship to cardiovascular and renal function. By employing WT and TNFR1‐KO mice and bone marrow transplantation studies, we established the critical involvement of TNFR1 signaling in the cardiac uptake of myeloid fibroblasts and concurrent development of fibrosis and hypertrophy (Duerrschmid et al. 2013, 2015). TNFR1 deficiency also prevented the increase in systolic blood pressure and worsening of cardiac function in response to 1 week infusion of Ang‐II (Duerrschmid et al. 2013). In the current study, after 2–4 weeks of Ang‐II exposure, we found that systolic blood pressure increased in TNFR1‐KO mice to levels seen in WT mice. Similarly, cardiac function deteriorated in these mice; at 6 weeks, hemodynamic parameters were not different than those in WT mice. By contrast, TNFR1‐KO hearts remained protected from the Ang‐II‐induced cardiac fibrosis, hypertrophy, and remodeling, suggesting that these responses were distinct from the Ang‐II‐induced hemodynamic changes.

In contrast to the heart, renal fibrosis developed slowly. After 6‐week Ang‐II infusion, when most inflammatory mediators and myeloid cells were absent in the heart, proinflammatory M1 and profibrotic M2 cells and their products appeared in the kidney and tubulointerstitial collagen deposition was observed. Notably, the development of renal fibrosis coincided with the upregulation of MCP‐1 and infiltration of monocytic CD34^+^ fibroblast precursors, which were absent at 1 week. Similar to the heart, TNFR1‐KO mice did not develop renal fibrosis, and neither were proinflammatory and profibrotic mediators elevated in the kidney. However, renal failure marker levels were equally elevated in both mouse groups, again suggesting that the functional effects of Ang‐II were distinct from its inflammatory and fibrotic effects, or that the degree of dysfunction induced by this treatment at 6 weeks was not measurable by the markers commonly used to measure renal function.

### Hypertension

Other groups demonstrated that general blockade of TNF with etanercept prevented the increase in blood pressure in genetically hypertensive and in Ang‐II‐infused rats (Elmarakby et al. 2006; Guzik et al. 2007), and that hypertension was blunted in 2‐ to 3‐week Ang‐II‐infused TNF‐deficient mice (Zhang et al. 2014a; Sriramula and Francis 2015). Other studies using TNFR1‐KO mice described an even greater hypertensive response to Ang‐II infusion than WT mice (Chen et al. 2010), or no difference between TNFR1‐KO and WT mice (Singh et al. 2013). In our Ang‐II studies, we observed no increase in systolic blood pressure (SBP) in TNFR1‐KO mice after 1‐week Ang‐II exposure (Duerrschmid et al. 2013), but SBP gradually increased after 2–4 weeks to levels similar to WT levels. We would like to point out that in our studies, we used Ang‐II stimulation only, whereas the two above‐mentioned groups using TNFR1‐KO mice supplemented Ang‐II infusion with a high‐salt diet. The addition of salt may be responsible for a faster increase in SBP and thus may account for the divergent outcomes. The mechanisms for the delayed raise in blood pressure in Ang‐II‐infused TNFR1‐KO mice were not addressed in this study, but may relate to reduced generation of reactive oxide species through decreased activation of vascular NAD(P)H oxidase and/or smaller changes in cell proliferation and extracellular matrix production in TNFR1‐deficient endothelial and smooth muscle cells (Ungvari et al. 2006; Lee et al. 2009).

### Cardiac anatomical and functional parameters

Similar to the slow increase in hypertension, hemodynamic changes in diastolic and systolic parameters of cardiac function in response to Ang‐II were also initially unaffected in TNFR1‐KO mice (Duerrschmid et al. 2013). However, they increased over the 6‐week Ang‐II infusion period to levels comparable with WT levels, indicating a slower, but steady worsening of cardiac function in TNFR1‐KO mice over time. Despite this, during the same time period, TNFR1‐KO mice developed only minor cardiac hypertrophy and remodeling, and no fibrosis, demonstrating that TNFR1‐signaling effected Ang‐II‐induced changes in anatomical parameters, but had little to no impact on functional parameters. These data also imply that Ang‐II‐mediated cardiac remodeling may act through different mechanisms than Ang‐II‐mediated cardiac dysfunction, and that one may develop, at least temporally, independent of the other. It will have to be tested whether the changes in anatomical parameters in TNFR1‐KO hearts will remain minor over longer Ang‐II infusion periods, or if they will eventually “catch up” to levels seen in the WT group, as seen for the changes in functional parameters. Also, the association between hypertension and cardiac remodeling remains to be characterized. In WT mice, being hypertensive for 1 week was long enough to associate with major development of cardiac hypertrophy, LV remodeling, and fibrosis (see also (Duerrschmid et al. 2013; Haudek et al. 2010)), whereas in TNFR1‐KO mice, being hypertensive for 2–3 weeks was not sufficient to significantly induce these changes.

### Cardiac fibrosis

Many reports in the literature describe an association between cardiac inflammation and fibrosis, and interventions that reduce the first also diminish the second, yet the mechanisms of this interaction are still unclear. Based on previous work, we propose that interstitial cardiac fibrosis arises from immune‐inflammatory dysregulation: We have shown that Ang‐II resulted in the upregulation of MCP‐1 within 1 day of infusion, which corresponded with the appearance of CD45^+^ monocytic cells that polarized into cytokine‐producing M1 cells. These cells, their products, and concurrently produced Th1‐related factors altogether created an initial proinflammatory milieu. After 3–7 days, under the influence of Th2‐related factor production, infiltrating monocytes became profibrotic M2 cells. This profibrotic environment drove the maturation of an M2 subpopulation further into fibroblasts that produced collagen (Duerrschmid et al. 2015). These mechanisms were MCP‐1 dependent, as monocytic fibroblast precursors and concurrent development of cardiac fibrosis were absent in hearts of mice deficient in MCP‐1 (Haudek et al. 2010). Further investigation using in vivo and in vitro models demonstrated the requirement for TNFR1 signaling in MCP‐1 activation, concurrent cardiac uptake of proinflammatory and profibrotic myeloid cells, and in monocyte‐to‐fibroblast polarization. Specifically, we described that TNF produced by M1 cells was involved in maturation of M2 cells to fibroblasts through TNFR1 signaling (Duerrschmid et al. 2013, 2015). By means of lineage‐tracing experiments using cells lacking TNFR1, we further demonstrated that monocytic fibroblast precursors were of bone marrow origin (Duerrschmid et al. 2015). With these experiments, we established a critical role for TNFR1, but not for TNFR2, signaling in the generation of fibroblast precursors and concurrent development of cardiac fibrosis in response to Ang‐II exposure.

The current report extends our observations by showing that the early Ang‐II‐induced inflammatory response, including the presence of myeloid M1 and M2 cells, dissipated over time. However, distinct fibrotic mechanisms were still activated. In particular, in WT hearts after 6‐week infusion, we found that collagen deposition was maintained at similar levels as observed after 1 week, yet proinflammatory TNF and MCP‐1 transcription was absent. Profibrotic gene expression, such as that of collagen types I and III, *α*‐SMA, and TGF‐*β*1, was still activated, albeit at lower levels than after 1‐week Ang‐II infusion. Importantly, TNFR1‐KO mice remained protected from the Ang‐II‐induced cardiac fibrosis, despite the increase in hypertension. These data confirmed our previous hypothesis that Ang‐II‐induced cardiac fibrosis was triggered by a rapid MCP‐1‐dependent uptake of myeloid precursors that mature into collagen‐producing fibroblasts, but that this response was transient and resolved quickly over time. As such, the progression and maintenance of cardiac fibrosis depended on other mechanisms independent of this cell population and not characterized in this study. The authors thus would like to emphasize again that the chemokine‐dependent mechanisms initiating cardiac fibrosis as described by our group may not be the only mechanism by which fibroblasts and their products are generated as part of growth or in response to pathological stimuli.

### Renal fibrosis

Renal tubulointerstitial fibrosis is characterized by excessive deposition of collagens and other extracellular matrix proteins in spaces between tubular nephrons, blood vessels, and the renal capsule, which results in the destruction of renal parenchyma and progressive loss of kidney function (Strutz and Muller 2006). However, the origins of activated fibroblasts in kidney remain unclear, as collagen‐producing cells may arise from resident cells, bone marrow‐derived cells, periadventitial cells, and by the process of epithelial‐to‐mesenchymal transition (Strutz and Muller 2006; Wada et al. 2011; Falke et al. 2015; Mack and Yanagita 2015).

We have previously described the uptake of chemokine‐dependent monocytic fibroblast precursors in an unilateral ureteral obstruction (UUO) model (Chen et al. 2011; Xia et al. 2014b), and in Ang‐II‐induced renal disease (Xu et al. 2011; Xia et al. 2013, 2014a). However, the latter studies involved Ang‐II infusion in combination with uninephrectomy to accelerate renal injury. The current study is thus different, insofar as we characterized the acute inflammatory and fibrotic responses due solely to elevated systemic levels of Ang‐II. This experimental setup may therefore account for the slow and modest development of renal fibrosis compared to our previous studies in which major development of fibrosis was observed after 4 weeks of infusion. The development of fibrosis was accompanied by an increase in MCP‐1 and TNF expression, as well by the concurrent presence of monocytic CD34^+^ fibroblast precursors, proinflammatory M1, and profibrotic M2 cells, the latter also producing collagen type I. The renal influx of distinct M1 and M2 cells was also described by our group and others following UUO (Braga et al. 2012; Yang et al. 2013; Zhang et al. 2014b).

Unlike our previous studies, however, the inflammatory response was not accompanied by an increase of CXCL16. This finding was unexpected, as we have previously observed a critical role for CXCL16 in UUO and Ang‐II/uninephrectomy‐induced renal injury (Chen et al. 2011; Xia et al. 2013). We suggest that CXCL16 augmented the response to Ang‐II. In contrast to MCP‐1 which was induced in the venular endothelium (Haudek et al. 2010), we have shown that CXCL16 was induced in tubular epithelial cells (Xia et al. 2013). The lack of CXCL16 induction in our model may also account for the more modest fibrosis; perhaps, both MCP‐1 and CXCL16 may augment cellular uptake and differentiation of myeloid fibroblasts. It is also possible that the CXCL16 response occurs only when the kidney has a direct parenchymal injury which induces its synthesis by renal tubules. Importantly, TNFR1‐KO kidneys remained protected from Ang‐II‐induced fibrosis. Thus, renal fibrosis was initiated by similar mechanisms as described for the heart, yet, due to unknown protective mechanisms, the development of fibrosis in the kidney was much slower and less prominent than observed in the heart.

Our results add to a body of literature suggesting a detrimental role for Ang‐II and TNF expression in the development of kidney fibrosis and failure (Mezzano et al. 2001; Ruiz‐Ortega et al. 2002; Esteban et al. 2004). Ang‐II was shown to exert its actions mainly through engagement of Ang‐II type 1 (AT1), which is highly expressed in kidney (Edwards and Aiyar 1993; Esteban et al. 2004). Both TNFR1 and TNFR2 are also expressed in glomeruli, and activation of intrinsic glomerular cells by soluble TNF requires TNFR1 signaling (Taubitz et al. 2013). The involvement of TNF and Ang‐II signaling in obstructive nephropathy was previously described (Guo et al. 1999, 2001). In these studies, the authors suggested that endogenously produced Ang‐II and TNF both contributed to the development of renal fibrosis through engagement of AT1 and TNFR1 and concurrent activation of transcription factor NF‐*κ*B and TGF‐*β*1 synthesis. However, the origin of activated fibroblasts was not identified in these studies (Guo et al. 2001). Another study employing 2‐week Ang‐II infusion together with high‐salt intake found that TNFR2‐KO mice were protected from the development of renal fibrosis and macrophage infiltration, whereas TNFR1‐KO mice were not (Singh et al. 2013). In our study, TNFR2‐KO mice developed renal fibrosis to a similar extent as WT mice, but TNFR1‐KO mice were protected from the Ang‐II‐induced fibrosis. The reasons for these contradicting results are unknown, but the difference in experimental procedures may be accountable.

## Conclusion

Mice deficient in TNFR1 remained protected from Ang‐II‐induced cardiac and renal fibrosis and cardiac hypertrophy after 6 weeks of infusion despite an increase in systolic blood pressure and decrease in cardiac and renal function. Fibrosis in the kidney developed more slowly, but was similarly associated with the uptake and maturation of myeloid fibroblast precursors as described for the heart. These results suggest that the inflammatory and fibrotic responses of Ang‐II are distinct from its effects on hemodynamic and cardiorenal function parameters with regard to the synergistic role of TNF signaling. Therefore, this study supplements previous work suggesting TNF synergy with Ang‐II, yet it is the first model to separate two pathological consequences of Ang‐II (inflammation/fibrosis and functional impairment) via the involvement of TNF synergy.

## Conflict of Interest

None declared.
